# VIDIIA Hunter: a low-cost, smartphone connected, artificial intelligence-assisted COVID-19 rapid diagnostic platform approved for medical use in the UK

**DOI:** 10.3389/fmolb.2023.1144001

**Published:** 2023-09-28

**Authors:** Aurore C. Poirier, Ruben D. Riaño Moreno, Leona Takaindisa, Jessie Carpenter, Jai W. Mehat, Abi Haddon, Mohammed A. Rohaim, Craig Williams, Peter Burkhart, Chris Conlon, Matthew Wilson, Matthew McClumpha, Anna Stedman, Guido Cordoni, Manoharanehru Branavan, Mukunthan Tharmakulasingam, Nouman S. Chaudhry, Nicolas Locker, Anil Fernando, Wamadeva Balachandran, Mark Bullen, Nadine Collins, David Rimer, Daniel L. Horton, Muhammad Munir, Roberto M. La Ragione

**Affiliations:** ^1^ Department of Comparative Biomedical Sciences, School of Veterinary Medicine, University of Surrey, Guildford, United Kingdom; ^2^ VIDIIA Ltd., Surrey Technology Centre, Guildford, United Kingdom; ^3^ Department of Microbial Sciences, School of Biosciences, University of Surrey, Guildford, United Kingdom; ^4^ Berkshire and Surrey Pathology Services, Molecular Diagnostics, Royal Surrey County Hospital, Guildford, United Kingdom; ^5^ Division of Biomedical and Life Sciences, Faculty of Health and Medicine, The Lancaster University, Lancaster, United Kingdom; ^6^ The Royal Lancaster Infirmary, University Hospitals of Morecambe Bay NHS Foundation Trust, Kendal, United Kingdom; ^7^ GB Electronics (UK) Ltd, Worthing, United Kingdom; ^8^ College of Engineering, Design and Physical Sciences, Brunel University London, Uxbridge, United Kingdom; ^9^ Centre for Vision, Speech and Signal Processing, University of Surrey, Guildford, United Kingdom

**Keywords:** rapid diagnostics, LAMP (loop mediated isothermal amplification), COVID-19, artificial intelligence, infectious diseases

## Abstract

**Introduction:** Accurate and rapid diagnostics paired with effective tracking and tracing systems are key to halting the spread of infectious diseases, limiting the emergence of new variants and to monitor vaccine efficacy. The current gold standard test (RT-qPCR) for COVID-19 is highly accurate and sensitive, but is time-consuming, and requires expensive specialised, lab-based equipment.

**Methods:** Herein, we report on the development of a SARS-CoV-2 (COVID-19) rapid and inexpensive diagnostic platform that relies on a reverse-transcription loop-mediated isothermal amplification (RT-LAMP) assay and a portable smart diagnostic device. Automated image acquisition and an Artificial Intelligence (AI) deep learning model embedded in the Virus Hunter 6 (VH6) device allow to remove any subjectivity in the interpretation of results. The VH6 device is also linked to a smartphone companion application that registers patients for swab collection and manages the entire process, thus ensuring tests are traced and data securely stored.

**Results:** Our designed AI-implemented diagnostic platform recognises the nucleocapsid protein gene of SARS-CoV-2 with high analytical sensitivity and specificity. A total of 752 NHS patient samples, 367 confirmed positives for coronavirus disease (COVID-19) and 385 negatives, were used for the development and validation of the test and the AI-assisted platform. The smart diagnostic platform was then used to test 150 positive clinical samples covering a dynamic range of clinically meaningful viral loads and 250 negative samples. When compared to RT-qPCR, our AI-assisted diagnostics platform was shown to be reliable, highly specific (100%) and sensitive (98–100% depending on viral load) with a limit of detection of 1.4 copies of RNA per µL in 30 min. Using this data, our CE-IVD and MHRA approved test and associated diagnostic platform has been approved for medical use in the United Kingdom under the UK Health Security Agency’s Medical Devices (Coronavirus Test Device Approvals, CTDA) Regulations 2022. Laboratory and *in-silico* data presented here also indicates that the VIDIIA diagnostic platform is able to detect the main variants of concern in the United Kingdom (September 2023).

**Discussion:** This system could provide an efficient, time and cost-effective platform to diagnose SARS-CoV-2 and other infectious diseases in resource-limited settings.

## 1 Introduction

At the end of 2019, a number of pneumonia cases of unknown aetiology, were detected in Wuhan City Hubei Province of China and reported to the World Health Organization (WHO) (WHO, 2020b). The causative agent, a new type of coronavirus, was identified in January 2020 as Severe Acute Respiratory Syndrome Coronavirus 2 (SARS-CoV-2) (Chan et al., 2020a; Zhou et al., 2020; Zhu et al., 2020). The SARS-CoV-2 is now the seventh type of coronavirus (within Coronaviridae family) known to cause infection in human. Four of these coronaviruses typically cause common cold symptoms (Su et al., 2016) and, before SARS-CoV-2, two other coronaviruses, both of zoonotic origin, were responsible for respiratory disease outbreaks, causing a number of casualties: the Severe Acute Respiratory Syndrome Coronavirus (now referred as SARS-CoV-1) in 2002-03 and the Middle East Respiratory Syndrome Coronavirus (MERS-CoV) in 2012-13 (Chan et al., 2015; Cui et al., 2019).

The coronavirus disease 2019 (COVID-19), caused by SARS-COV-2, rapidly became a pandemic (WHO, 2020c), and has infected more than 690 million individuals worldwide between December 2019 and September 2023, according to Worldometer (https://www.worldometers.info/coronavirus/1st September 2023); due to high incidence of person-to-person transmission and the existence of asymptomatic or mild symptomatic SARS-CoV-2 carriers (WHO, 2020a; Chan et al., 2020b; Liu et al., 2020). The COVID-19 pandemic has had significant social and economic impacts worldwide (Borio, 2020; Gu et al., 2020; Rogers et al., 2020; Swinnen and McDermott, 2020; Yang et al., 2020; Canas et al., 2021; Tangcharoensathien et al., 2021; Ozili, 2022). By identifying asymptomatic carriers and infected individuals at early stages, rapid and accurate diagnostics are key to control the spread of COVID-19, when paired with an effective track and tracing surveillance system (Peiris et al., 2003; Brouard et al., 2020; Olalekan et al., 2020; Peck, 2020; Walsh et al., 2020; Woo et al., 2020; Huff, 2021; Jones et al., 2021).

Immediately following the publication of the genome sequence of SARS-CoV-2 (Peck, 2020), reverse transcription real-time quantitative polymerase chain reaction (RT-qPCR) assays were developed, to recognise different target genes in SARS-CoV-2, such as the replicase complex (ORF1ab), spike (S), envelope (E), membrane (M), nucleocapsid (N) and RNA-dependent RNA polymerase (RdRP) genes (Peck, 2020; Vogels et al., 2020). RT-qPCR is recognised as the gold standard for COVID-19 diagnostics, due to its high sensitivity and specificity. However, because this type of assay requires a long reaction time (around 2 h), sophisticated instruments, and highly trained personnel (Babiker et al., 2020; Yu et al., 2022), diagnostics labs have not been able to fulfil the unprecedentedly high level of testing requests.

To reduce the time to diagnosis, assays using other types of Nucleic Acid Amplification Test (NAAT) technique, such as reverse transcription loop-mediated isothermal amplification (RT-LAMP), have been developed for the detection of SARS-CoV-2 (Ben-Assa et al., 2020; Chow et al., 2020; Jiang et al., 2020; Thompson and Lei, 2020). RT-LAMP only requires single amplification temperature, thus cutting the need for expensive thermal cycling instruments and results are available in 30 min or less (Hassan et al., 2022). In RT-LAMP assays, a set of six primers recognising eight distinct sites of the target sequence ensure high sensitivity and specificity (Wang et al., 2015; Hardinge and Murray, 2019; Hassan et al., 2022). Different methods of detection can be used to interpret the results of RT-LAMP assays including turbidimetry, fluorescence signal or colorimetry using pH-sensitive dyes such as phenol red or neutral red (Cui et al., 2020; Huang et al., 2020; Rohaim et al., 2020; Woo et al., 2020; Alves et al., 2021; Aoki et al., 2021; Garcia-Venzor et al., 2021; Lim et al., 2021). The RT-LAMP assays developed for SARS-CoV-2 detection have shown sensitivity and specificity comparable to RT-qPCR (Baek et al., 2020; Cui et al., 2020; Ganguli et al., 2020; Huang et al., 2020; Lamb et al., 2020; Mautner et al., 2020; Mohon et al., 2020; Park et al., 2020; Rodel et al., 2020; Rohaim et al., 2020; Yan et al., 2020; Fowler et al., 2021; Garcia-Venzor et al., 2021; Lalli et al., 2021; Lim et al., 2021; Nawattanapaiboon et al., 2021; Wei et al., 2021; Marino et al., 2022; Papadakis et al., 2022; Yu et al., 2022).

In this study, we developed further [from (Rohaim et al., 2020; Tharmakulasingam et al., 2021)] a SARS-CoV-2 rapid diagnostics platform that relies on a RT-LAMP colorimetric assay and a portable smart diagnostic device. The designed AI-assisted diagnostics solution recognises the N gene of SARS-CoV-2 with high clinical sensitivity and specificity. The sensitivity ranged from 98% (94.28%–99.32%) to 100% (97.50%–100%), depending on samples viral load and with a limit of detection of 3 copies of RNA per µL in 20 min and of 1.4 copies of RNA per µL in 30 min. The specificity was validated as 100% (97.50%–100%). Laboratory and *in silico* data presented here also indicates that the VIDIIA Hunter diagnostic platform is able to detect the main variants of concern in the United Kingdom (September 2023). Based on clinical validation in two NHS sites, the diagnostic platform has been approved for medical use in the United Kingdom, under the United Kingdom Health Security Agency’s Medical Devices (Coronavirus Test Device Approvals) Regulations 2022 (CTDA).

## 2 Materials and methods

### 2.1 Ethics approval statement

Clinical samples used to develop and validate the VIDIIA Hunter diagnostic platform were collected from two NHS sites: Royal Lancaster Infirmary, Lancaster and the Berkshire and Surrey pathology services, Surrey, United Kingdom.

The use of human tissue samples, collected from hospital patients, for research purposes is regulated by the Health Research Authority (HRA) for NHS Research and Ethics Committee (REC) approval. To validate the VIDIIA Hunter platform, human naso-pharyngeal swab samples were collected from the Berkshire and Surrey Pathology Services under HRA NHS REC ethics approval through the Integrated Research Application System (IRAS), IRAS project ID: 283201, NHS REC number: 20/EE/0125.

Clinical samples were also collected from the Royal Lancaster Infirmary and processed according to the guidelines and approval of the Faculty of Health and Medicine Research Ethics Committee (FHMREC) of Lancaster University, United Kingdom (under ID FHMREC19112). Approval was also sought from Integrated Research Application System (IRAS) which is available under IRAS project ID: 293291, and NHS REC Number: 21/EM/0078.

### 2.2 *In silico* nucleotide sequence comparisons and primer design

The Global Initiative on Sharing Avian Influenza Data (GISAID) was accessed on 27/03/2022, and 1,654 SARS-CoV-2 genomes were downloaded. Using the Wuhan-Hu-1 reference sequence (NC045512), the DNA translation of each ORF in the genome (ORFab1, S gene, ORF3a, M gene, E gene, ORF6, ORF7a, ORF7b, ORF8, N gene, and ORF10) were incorporated into a custom BLAST database. This database was used to query all 1,654 genomes from GISAID using the Abricate version 0.9.8. BLAST shell. Nucleotide variation and conservation of each ORF was determined. The N gene was determined to have a high degree of nucleotide conservation amongst all genomes.

LAMP primers targeting the N gene were designed using Primer Explorer V5 (Copyright Fujitsu Limited 1999–2005; http://primerexplorer.jp/e). A total of 3 sets of 6 primers were designed targeting 8 distinct regions of the gene: outer primers (F3 and B3), inner primers (FIP and BIP) and loop primers (LoopF and/or LoopB). The most appropriate set of primers was chosen, after preliminary experiments using synthetic RNA.

### 2.3 Clinical sample processing

To develop and validate the VIDIIA Hunter platform using clinical samples and data, a total of 630 nasal-pharyngeal swabs, collected in virus transport media (VTM) from patients as per standard NHS protocol (for COVID-19 testing) and tested using TaqPath™ COVID-19 assay (Thermo Fischer Scientific, Waltham, United States) by the Berkshire and Surrey pathology services at the Royal Surrey County Hospital, were anonymised using a unique (UoS) identifier before being transferred to the School of Veterinary Medicine at the University of Surrey. Out of the 630 clinical samples, 302 were detected COVID-19 positives and 328 negatives, between March 2020 and December 2021. At the School of Veterinary Medicine, RNA extraction was performed on 100 µL of VTM using the commercial QIAgen RNeasy kit (Qiagen, Valencia, CA, United States), according to manufacturer instructions. A total of 122 additional clinical samples were collected at the Royal Lancaster Infirmary from COVID-19 suspected patients through the routine NHS collection procedure for COVID-19 screening. These samples were stored and transported in the virus transport media (VTM) to the NHS diagnostic laboratory at Lancaster University, United Kingdom. Total RNA was extracted using 140 µL of the VTM by the commercial QIAamp Viral RNA Mini kit (Qiagen, Valencia, CA, United States).

For the final validation of the VIDIIA Hunter platform, a total of 150 positive clinical samples covering a dynamic range of clinically meaningful viral load: 60 samples with Ct values 25 and under, 60 samples with Ct values between 25 and 30 samples with Ct values above 30 as well as 250 negative samples were collected at the Berkshire and Surrey pathology services COVID-19 Lighthouse Laboratory (Bracknell) between December 2021 and April 2022. At the School of Veterinary Medicine, RNA extraction was performed on 100 µL of VTM using the commercial QIAgen RNeasy kit (Qiagen, Valencia, CA, United States), according to manufacturer instructions. Each sample was tested in duplicates.

### 2.4 Cloning and *in vitro* transcription of SARS-CoV-2 nucleocapsid target gene

DNA fragments containing SARS-CoV-2 nucleocapsid gene as gBlocks (2019-CoV Plasmid control DNA, Integrated DNA Technologies, United States) were transformed into DH10β *E. coli* chemically competent cells. After propagation and plasmid purification using the Qiagen Miniprep kit., the linearized plasmid was used for *in vitro* transcription of SARS-CoV-2 nucleocapsid gene, after addition of T7 RNA polymerase promoters by PCR and using the PCR products as templates. After RNA purification using RNA clean up columns (G-50) and determination of the RNA concentration by spectrophotometry (Biodrop, Biochrom LTD., United Kingdom), the RNA stock solution was adjusted to 100 ng/μL in Tris-EDTA buffer, and 10-fold serial dilutions were performed from 10 ng/μL to 0.01 fg/μL. This synthetic RNA was used as positive control for the RT-LAMP assays.

### 2.5 Real-time RT-PCR assay

All the samples (clinical and synthetic) used for the validation of the VIDIIA Hunter diagnostics platform were also tested using the CDC 2019-Novel Coronavirus (2019-nCoV) Real-Time RT-PCR Diagnostic Panel (Centers for Disease Control and Prevention, Division of Viral, Atlanta United States), containing the 2019-nCoV_N1, 2019-nCoV_N2 and Human RNase P combined primers and probes mix, as per the Instructions for Use (FDA.gov). The 25-µL RT-qPCR reactions consisted of 12.5 µL 2X Reaction Mix, 0.5 µM of each primer, and 0.2 µM probe, 0.5 µL of SuperScript^®^ III RT/Platinum^®^ Taq Mix, and 2 µL of RNA (extracted from clinical samples or synthetic). The amplification process was performed in the CFX96 Touch Real-Time PCR Detection System (BioRad Laboratories, Watford, United Kingdom), according to the recommended cycling protocol. The amount of viral RNA in each sample was estimated by comparing the cycle threshold values (Ct) to the standard curve made by serial 10-fold serial dilutions of synthetic RNA.

### 2.6 RT-LAMP assays

The 20 μL RT-LAMP reactions were prepared by mixing 10 μL WarmStart^®^ Colorimetric LAMP 2X Master Mix with UDG (M1804, New England Biolabs, United Kingdom), 2 μL of 10X primer mix (16 μM of FIP and BIP, 2 μM of F3 and B3, 4 μM of LF and LB), 1.6 μL of 400 mM Guanidine hydrochloride (G3272, Merck, Germany), 2.4 μL of DEPC-Treated Molecular Biology Grade water (CAS 7732-18-5—Calbiochem, Merck, Germany) and 4 μL of sample. The primers sequences (5′-3′) were as follows: F3: TCCTGCTAACAATGCTGC - B3: TCT​CAA​GCT​GGT​TCA​ATC​TG - FIP: AAC​GAG​AAG​AGG​CTT​GAC​TGC​TCA​AGG​AAC​AAC​ATT​GCC​A - BIP: CTC​ATC​ACG​TAG​TCG​CAA​CAG​TAT​TGC​CAG​CCA​TTC​TAG​C - LoopF: CCTTCTGCGTAGAAGCCTT–LoopB. AAT​TCA​ACT​CCA​GGC​AGC​A. A negative no template control (NTC) and a positive control: synthetic RNA with a fixed concentration of 0.1 ng/μL were included in every run. Amplification was performed and monitored using the VH6 Device.

### 2.7 Automated image acquisition and processing through edge detection

**FIGURE 1:**
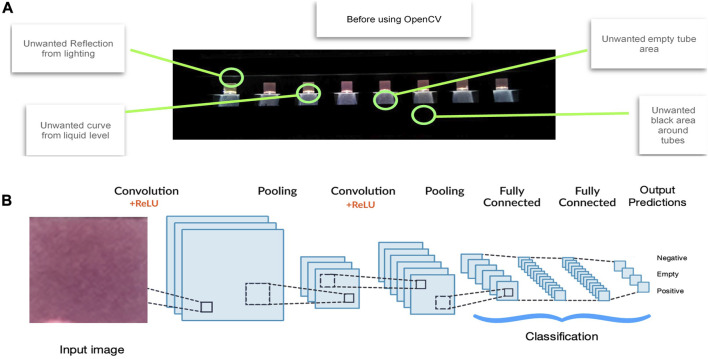
Artificial Intelligence method. VIDIIA’s AI solution works in two stages: **(A)** Tube identification process. The tubes in the image are identified by using an edge detection method (OpenCV) that removes all the unwanted regions such as the empty tube areas, black areas around the tubes, reflection form lighting and curves from liquid levels that could mislead the Artificial Intelligence model. **(B)** Deep Learning process feature classification. By using over 10,000 images, and growing, as training data, our two-dimensional Deep Learning model (using convolutional neural network) classifies the input images into any of the specified categories: Negative, Positive and Empty. The overall training process of the Convolution Network may be summarized as follows. The filters and parameters/weights are first initialised with random values. The network then takes a training image as input, goes through the forward propagation step (convolution, ReLU and pooling operations along with forward propagation in the Fully Connected layer) and finds the output probabilities for each class. The total error at the output layer is calculated with the following formula: Total Error = ∑ ½ (target probability–output probability) ^2^. Backpropagation is used to calculate the gradients of the error with respect to all weights in the network and use gradient descent to update all filter values/weights and parameter values to minimize the output error. The weights are adjusted in proportion to their contribution to the total error. When the same image is input again, output probabilities might now be closer to the target vector. This means that the network has learnt to classify this particular image correctly by adjusting its weights/filters such that the output error is reduced. Parameters like number of filters, filter sizes, architecture of the network are all fixed before the training process is repeated with all images in the training set; until the model classifies the images correctly every time.

The LAMP assay in 8 separate tubes was initiated remotely to start heating to 66.2°C, resulting images were then captured using the inbuilt RPi Camera (every 20 s) and were saved in the RPi in the RGB colour space and lossless portable network graphics (PNG) formats. The tubes and the images were identified by using an edge detection method (OpenCV) (Chen et al., 2020) that removes all the unwanted regions such as the empty tube areas, black areas around the tubes, reflection form lighting and curves from liquid levels that could mislead the Artificial Intelligence model (Figure 1A). After this process the images were then passed onto the AI model for analysis.

### 2.8 Artificial intelligence based, test-tube features detection

Artificial Intelligence (AI), deep learning model, can identify hidden patterns (features), in a given training data set, which has been validated and manually selected by LAMP experts. The training data set contained 5,000 tube images in the “negative” category, 5,000 tube images in the “positive” category and 5,000 tube images in the “empty” category. These myriads of images were extracted from different trials across different devices in different labs, using the 752 clinical samples. This training data set was then passed through a preparation process before training the deep learning model, which is based on Convolutional Neural Networks.

A 2-dimensional CNN (Sharif et al., 2021) architecture was proposed with a 2-dimensional pooling (Figure 1B) and this architecture allowed a lightweight model with enough detail to train the deep learning layer. For the training of the network, the dataset was shuffled and then split into an 8.5:1.5 proportion. The 85% of the data was used to train the network and the remaining 15% was exploited to check how the network behaves when it identifies an image that it has not seen before. As training a dataset requires loading numerous images in each single operation, this methodology was too memory intensive for our product. Therefore, a data generator was implemented which reads the data in batches from the dataset directory and transfers it to the model. After multiple experiments, it was observed that the network converged after 10 cycles (epochs) through the dataset. This 10-epoch cycle was subsequently adopted throughout to decrease the probability of overfitting. In addition, an additional set of 1,000 test-tube crops were used to validate the network. The best performing network resulted in an accuracy of 98% in classifying tubes based on their colours.

This approach helps to overcome different variances that could affect their automated interpretation such as: brightness, contrast, external lighting, air exposure of reagents, sample incubation time, occlusion, intraclass variation, bubbles, variables pH in human samples. To assess the temporal impact of the AI-assisted detection of positive, negative, or empty features, RT-LAMP reaction was run with 3 known positive and negative patient samples as well as positive and negative controls. We assessed the changes in the colour after every 5 min until complete stoppage of the LAMP reaction at 30 min. The gradual colour changes were detectable with naked eye as early as 20 min post-start of the reaction. Corresponding samples were run on the newly developed device and temporal and real time colour changes were monitored as described earlier. A clear change in the colour was calibrated to be as early as 15 min using device operated processing of the data.

### 2.9 Analytical specificity and limit of detection of the VH6 diagnostic

The designed and selected N gene LAMP primers set was validated for its analytical specificity by testing cross-reactivity against other respiratory pathogens: Adenovirus Type 6, *Bordetella pertussis* and *parapertussis, Chlamydia pneumoniae*, Coronaviruses including 229E HKU1, NL63, OC43 surrogates, Human Metapneumovirus surrogate, Human Rhinovirus, Influenza A (subtypes H1, H1-2009 and H3) and Influenza B, *Mycoplasma pneumoniae*, Parainfluenza Viruses (1, 2, 3, and recombinant 4a) and Respiratory Syncytial Virus (using the Respiratory (21 Targets) Control Panel, Microbiologics Minnesota, United States). The limit of detection of the developed RT-LAMP assay was also evaluated by testing serial dilutions of the EDX SARS-CoV-2 Positive Run Control (#COV019CE, Biorad, Watford, United Kingdom), which contains synthetic RNA transcripts of five SARS-CoV-2 gene targets (E, N, ORF1ab, RdRP and S Genes, 200,000 copies/mL or 200 copies/µL for each gene) and Human Genomic DNA (75,000 copies/mL). The control was diluted to obtain 140, 14, 3, and 1.4 copies/µL and each dilution was tested with the VIDIIA Hunter platform.

### 2.10 Evaluation of test accuracy

Following validation, testing of the 400 clinical samples, contingency tables were constructed and clinical sensitivity with 95% 2-sided confidence interval, clinical specificity with 95% 2-sided confidence interval positive and negative predictive values were calculated, using the following formulae:

$$Sensitivity=\left[\frac{positives\;with\;both\;LAMP\;and\;RTqPCR}{positives\;using\;RTqPCR}\right]\times 100$$

$$Specificity=\left[\frac{negatives\;with\;both\;LAMP\;and\;RTqPCR}{negatives\;using\;RTqPCR}\right]\times 100$$

$$Lower\;95\%\;CI=\frac{A-B}{C}\times 100\;Upper\;95\%\;CI=\frac{A+B}{C}\times 100$$

where $A=2r+1.96^{2}\;B=1.96\;\sqrt{1.96^{2}+4r\left(1-p\right)}\;C=2\left(n+1.96^{2}\right)$

*r* = number of true positives (for sensitivity) or number of true negatives (for specificity)

*n* = number of positives on comparator RT-qPCR (for sensitivity) or number of negatives on the comparator RT-qPCR (for specificity)

*p = r/n* (i.e., sensitivity or specificity as a proportion)

$$Positive\;predictive\;value\;\left(PPV\right)=\left[\frac{positives\;with\;both\;LAMP\;and\;RTqPCR}{total\;of\;positive\;tests\;with\;LAMP}\right]\times 100$$

$$Negative\;predictive\;value\;\left(NPV\right)=\left[\frac{negatives\;with\;both\;LAMP\;and\;RTqPCR}{total\;of\;negative\;tests\;with\;LAMP}\right]\times 100$$

### 2.11 Variants of concern testing

The VIDIIA Hunter platform was also validated with chemically inactivated standards of the alpha variant SARS-CoV-2/UK/VUI/1/2020 (Public Health England, Wiltshire, United Kingdom), the beta variant SARS CoV-2/South Africa/VOC202012/02 (Public Health England, Wiltshire, United Kingdom) and the gamma variant SARS CoV-2/Brazil/P1 (Public Health England, Wiltshire, United Kingdom).

The VIDIIA Hunter platform was used throughout the pandemic alongside RT-qPCR (between March 2020 and April 2022) in collaboration with the Berkshire and Surrey Pathology Services (Royal Surrey County Hospital and Lighthouse lab). During this period, several SARS-CoV-2 variants of concern (VOCs) were detected in clinical nasal-pharyngeal swab samples collected from COVID-19 suspected patients through routine NHS collection procedure. The VOCs were identified by RT-qPCR (SNPsig^®^ SARS-CoV-2 Es-capePLEX, by Primerdesign Ltd., Chandler’s Ford, United Kingdom) and through independent sequencing, as per government requirements. According to the United Kingdom Health Security Agency (https://www.gov.uk/government/publications/covid-19-variants-genomically-confirmed-case-numbers, 1st September 2023) and the GISAID initiative data (https://gisaid.org/hcov19-variants/, 1st September 2023):

• Alpha SARS-CoV-2 VOC was predominant between December 2020 and May 2021, with some Beta and Gamma SARS-CoV-2 VOC between March and May 2021
• Delta SARS-CoV-2 VOC was predominant between June and November 2021
• Omicron SARS-CoV-2 VOC was predominant between December 2021 and April 2022

The test is currently subjected to monthly VOCs assurance surveillance. This is done *in silico*, by comparing the RT-LAMP primer sequences against the variant sequence submissions within the GISAID database (https://gisaid.org/, 1st September).

### 2.12 VIDIIA Hunter Diagnostics Platform process

**FIGURE 2:**
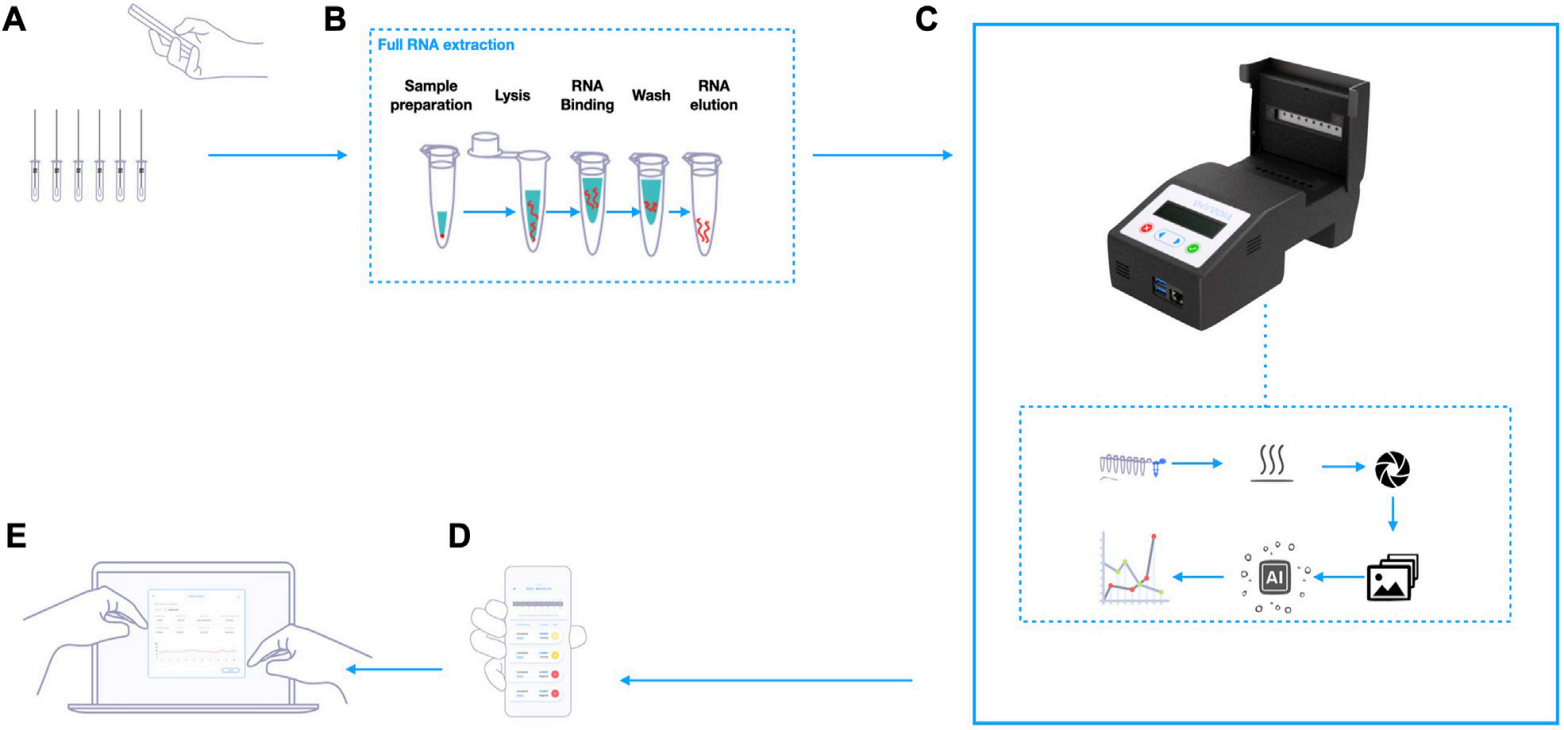
VIDIIA Hunter Diagnostics Platform process overview. **(A)** Using the VIDIIA companion app, the operator scans the unique identifiers attributed to nasal-pharyngeal swabs samples, that are collected in Virus transport media (VTM). **(B)** Samples are processed by full RNA extraction, using a commercial kit. **(C)** After preparing the RT-LAMP reactions and transferring the samples, according to VIDIIA’s instructions, the reaction tubes are inserted in the VH6 device. The VH6 device heats up the reaction tubes, for amplification and takes pictures throughout the amplification. At the end of the amplification, an artificial intelligence (deep-learning model) analyses the pictures and outputs the results. **(D)** The VIDIIA companion app connects to the device and retrieves the results from the VH6, displays them and uploads them to VIDIIA cloud. **(E)** VIDIIA users’ friendly online dashboard can then be used to consult and analyse data.

The complete workflow from samples collection to the AI-assisted results using the VIDIIA Hunter platform is shown in Figure 2. First, nose and throat swab samples were collected from the patient and transferred into a collection tube containing Virus transport media (VTM). Using the VIDIIA companion app (smartphone application for Android, trademark of Google LLC), the operator scanned the unique identifiers attributed to nasal-pharyngeal swabs samples. Then, a full RNA extraction was performed on the VTM, using a commercial kit (QIAgen RNeasy) (Figure 2). Next, 4 µL of the extracted samples and positive control (N gene synthetic RNA) were transferred respectively into tubes 2 to 7 and tube 8 of an 8-strip PCR tubes containing 16 µL of the RT-LAMP reagents (Figure 2). For negative control, 20 µL of the RT-LAMP reagents were transferred into the first tube of the 8-strip PCR tubes. The 8-strip PCR tubes was then transferred into the VH6 device, controlled by the VIDIIA companion app that registers patients samples and manages the entire process. The super mix contains phenol red to monitor pH change (through colour change) due to the nucleic acid amplification which occurs at 66.2°C, in presence of viral RNA (Figure 2). These changes were monitored in the VH6 using a camera, which takes pictures throughout the amplification. These images were then passed to an AI deep learning model for their analysis and to output diagnostics results. The application retrieved the results and stored them in the cloud. The administrator’s account was able to access, manage and release the results to their respective patients (Figure 2).

## 3 Results

### 3.1 Manufacture of an isothermal nucleic acid amplification device with deep learning model for features detection

A device was designed and manufactured using off-the-shelf electronic components, assembled on a custom-designed printed circuit board (PCB). It included customised flexible resistive heating elements (5W, NEL, UK), and specially designed aluminium heating blocks. Raspberry Pi (RPi, trademark of Raspberry Pi Ltd.) was used to control the main functionality of the device (heating control, image capture and communications) with an ATSAM supervisor microcontroller to provide real-time functionality and a watchdog to ensure the software is functioning throughout. The thermal profiling was designed and optimised to ensure the stable management of the assay whilst performing the LAMP reaction. This included an array of digital temperature sensors (DS18B20, Maxim Integrated, United States) positioned directly on the PCB boards to monitor heater block temperature changes and provide feedback control. The temperature was controlled by hardware, firmware and fine-tuned within the RPi software. The aluminium heater block was designed to hold a strip of 200 µL PCR tubes, with a lid heater to prevent condensation. They were attached directly on top of the surface mount temperature sensors on the respective PCBs with a heat transferring adhesive (TermoGlue, Termopasty Grzegorz Gasowski, Poland). The flexible resistive heating elements were also attached to the heater blocks. To circumvent the need for specialised docks and eliminate user interpretation of the colorimetric results, a Raspberry Pi Camera (RPi Camera) was used. Eight LEDs (LW T733, Osram, Germany) were assembled on the top side of the lid mount PCB to shine light directly into the reaction tubes to achieve consistent lighting within the device. All components were assembled into a custom-designed enclosure (14.3 × 10.8 × 6 cm) with openings to access to the USB and TCP/IP ports of the RPi. The user-interface (UI) was via an OLED screen and membrane keypad. A 20,000 mAh power bank (Anker Power Core, Anker, China) with two 5V, 2A output was used to power the device. A Python based control software was used to control the heating, image the progression of the LAMP assay and store the “time-lapse” images and temperature data within the device’s memory. The user can initiate a test by either connecting the VH6 device via the VH6 Wi-Fi access point or via the VH6 Ethernet port, with a host device (PC, mobile phone, tablet) to the internal web server portal running on the RPi to set-up and operate the unit or through simply pairing the device with the mobile app via wireless communication and selecting the required settings. The OLED screen displays the status of the device throughout a test, a timer and the status of the test are also displayed on the VIDIIA companion app, if the smartphone application is being used.

### 3.2 Artificial intelligence-assisted features detection associated with the RT-LAMP reaction

**FIGURE 3:**
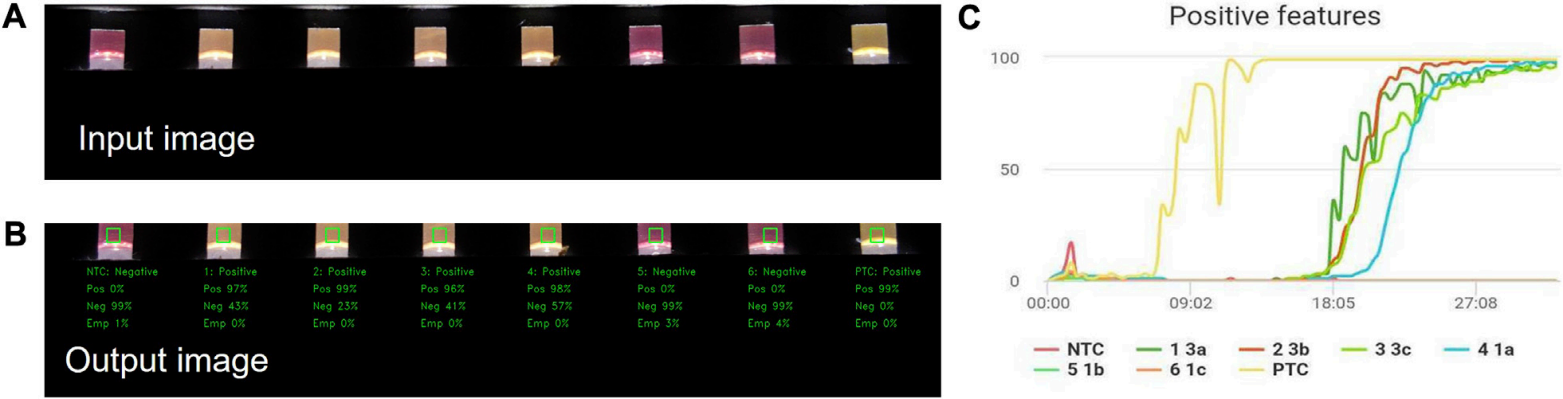
Artificial Intelligence results display. The VH6 software retrieves the images taken during the experiment and pass them through the inbuilt Artificial Intelligence (AI) model to obtain the predictions (Positive, Negative and Empty features); this information is then retrieved by the VIDIIA mobile app and displayed to the user showing the **(A)** input image, **(B)** output image with the probabilities of features (Positive, Negative and Empty) and the **(C)** “Positive features” graph created using the Predicted features of each tube calculated every minute until the end of the experiment.

After being trained, with a 15,000 tube images dataset, to identify positive, negative and empty features, the 2-dimensional Convolutional Neural Networks (CNN) AI model was used to run a full analysis of the input image (Figure 3A), quantify the features detected in each tube for each of the target categories (Positive, Negative and Empty) and assigned a percentage value per category. The model then weighted the percentage values generated per tube to label the sample results according to the highest percentage. The percentages of features and labels for each tube were then displayed on the output image linked to the VIDIIA cloud and the companion app (Figure 3B).

The output data from the AI was then passed to an upper layer interface through the VH6 firmware in a form of an API (Application Programming Interface). The VIDIIA companion app uses the data from the API and then presents the results to the user and plot a graph (Figure 3C). The graph is built by analysing, through the AI, every image taken by the device (every 20 s) within the time of the experiment. The positive predicted features were considered to build the chart; it is possible to plot other graphs based on the other target categories (Delta: Positive-Negative, Negative or Empty) however the more comparative graphs to RT-qPCR are the positive and delta features graph.

Furthermore, the AI has an error coping mechanism able to deal with issues that may occur during a test:

• If the positive control (PTC) shows a high percentage of negative features (more than positives) or if all the tubes change colour (including the negative control), the AI will output a VOID error code.
• If the tubes do not show a prevalence on any of the predicted features (Positive, Negative and Inconclusive), above 5%; the AI will output an Inconclusive state.
• If the negative control tube shows a high percentage of positive features (more than negatives), the AI will output a Warning error code.
• If the operator does not insert a sample, the AI will output an Empty tube state on tubes that show a high percentage of empty features.

### 3.3 Limit of detection and analytical specificity of the VIDIIA hunter diagnostic platform

**FIGURE 4:**
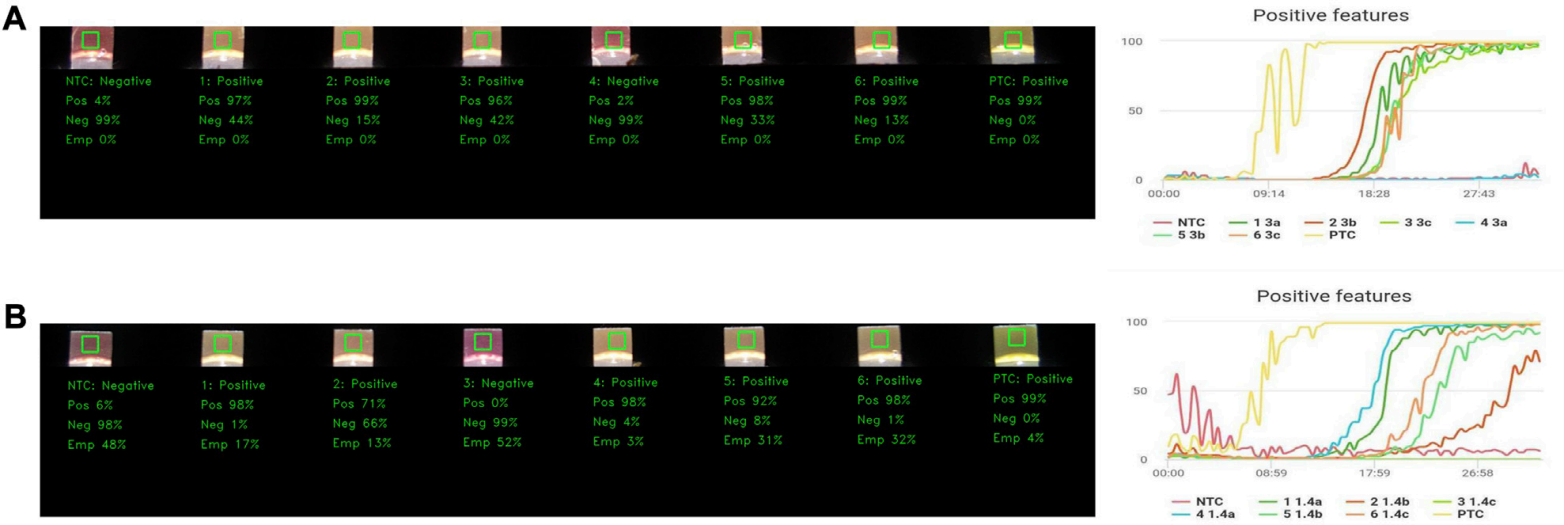
Limit of detection of the VIDIIA Hunter diagnostic platform. The limit of detection of the VIDIIA Hunter was evaluated by testing different concentrations of the EDX SARS-CoV-2 Positive Run Control (Biorad, Watford, United Kingdom): 140, 14, 3, and 1.4 copies/µL. **(A)** Results obtained with a concentration of 3 copies of RNA/µL. A test was prepared with a negative control (NTC) in tube 1, 6 samples containing 3 copies of RNA/µL in tubes 2 to 7 and a positive control (PTC) in tube 8. After 30 min of amplification using the VIDIIA Hunter platform, the output image and positive features graph shows that 5 out of 6 samples containing 3 copies of RNA/uL are positives for SARS-CoV-2, the positive control is also positive and the negative control negative. The graph shows that the RNA started to be amplified between 15 and 20 min. **(B)** Results obtained with a concentration of 1.4 copies of RNA/µL. A test was prepared with a negative control (NTC) in tube 1, 6 samples containing 1.4 copies of RNA/uL in tubes 2 to 7 and a positive control (PTC) in tube 8. After 30 min of amplification using the VIDIIA Hunter platform, the output image and positive features graph shows that 5 out of 6 samples containing 1.4 copies of RNA/µL are positives for SARS-CoV-2, the positive control is also positive and the negative control negative. The graph shows that the RNA started to be amplified between 15 and 27 min.

The limit of detection (LoD) was evaluated by testing serial dilutions of the EDX SARS-CoV-2 Positive Run Control (#COV019CE, Biorad, Watford, United Kingdom), which contains 200,000 copies/mL (200 copies/µL) of synthetic RNA transcripts of five SARS-CoV-2 gene targets (E, N, ORF1ab, RdRP and S) and 75,000 copies/mL of Human Genomic DNA. Different control concentrations were tested with the VIDIIA Hunter diagnostic platform: 140, 14, 3 and 1.4 copies/µL. Our diagnostic test, using the EDX SARS-CoV-2 Positive Run Control, detected as low as 3 copies of RNA/µL with an 81.57% detection rate (31 out of 38 replicates) and 1.4 copies of RNA/µL with a 68.42% detection rate (26 out of 38 replicates) (Table 1). For samples at a concentration of 3 copies/µL, positive features were detected by the AI between 15 and 20 min whilst the positive control showed positive features in less than 10 min (Figure 4A). For samples at a concentration of 1.4 copies/µL, positive features were detected by the AI between 18 and 28min whilst the positive control showed positive features in less than 10 min (Figure 4B).

**TABLE 1:** Limit of detection of the VIDIIA Hunter platform. The limit of detection of the VIDIIA Hunter was evaluated by testing different concentrations of the EDX SARS-CoV-2 Positive Run Control (Biorad, Watford, United Kingdom): 140, 14, 3, and 1.4 copies/µL. Detection rates were calculated by dividing the number of detected samples by the number of tested samples.

RNA concentration	Tested samples	Detected samples	Detection rate (%)
140 copies of RNA/µL	31	31	100
14 copies of RNA/µL	31	31	100
3 copies of RNA/µL	38	31	81.57
1.4 copies of RNA/µL	38	26	68.42
1 copy of RNA/µL	9	3	33.33
0.3 copies of RNA/µL	3	0	0

To evaluate the analytical specificity, the designed N gene LAMP primers set was used to test cross-reactivity against 21 other respiratory pathogens, including other Coronaviruses, by using a Respiratory (21 Targets) Control Panel (Microbiologics Minnesota, United States). No amplification was observed for any of the other respiratory pathogens, showing an analytical specificity of 100%.

### 3.4 Validation of VIDIIA hunter method on extracted clinical samples and diagnostic accuracy

To develop and validate the VIDIIA Hunter platform and its associated RT-LAMP assay, RNAs were extracted from 752 clinical samples, collected at the Royal Surrey County Hospital (RSCH) and the Royal Lancaster Infirmary of the National Health Service (NHS) and tested in parallel using the VIDIIA Hunter platform and the CDC 2019-Novel Coronavirus (2019-nCoV) Real-Time RT-PCR Diagnostic Panel (as reference method). The RT-qPCR assay detected a total of 367 positive and 385 negative samples in a cohort of 752 patients’ samples and the VIDIIA Hunter platform detected 364 positive and 388 negative samples, with 7 samples giving different results (5 were positive with RT-qPCR, but negative with RT-LAMP and 2 were negative with RT-qPCR, but positive with RT-LAMP). This data facilitated the validation of the RT-LAMP assay and device’s settings and was used to train the AI model.

For the final clinical validation of the VIDIIA Hunter platform, the RT-qPCR assay (performed at a regional Lighthouse lab) detected a total of 150 positive clinical samples covering a dynamic range of clinically meaningful viral load: 60 samples with Ct values 25 and under (high viral loads), 60 samples with Ct values between 25 and 30 (medium viral loads), and 30 samples with Ct values above 30 (low viral loads); and 250 negative samples. Each sample was tested in duplicates. The VIDIIA test and Hunter platform detected 147 positive samples and 253 negative samples (Table 2; Supplementary Table S1). All 60 samples with high viral loads (Ct values 25 and under) were detected positive using both RT-qPCR and the VIDIIA Hunter platform (Supplementary Table S1). Out of the 90 samples with medium and low viral loads [Ct values between 25 and 36.02 (N gene)], 3 samples were positive by RT-qPCR but negative with the VIDIIA Hunter platform (Ct values of 25.15, 29.38 and 36.02 for the N gene Supplementary Table S1).

**TABLE 2:** Validation of the VIDIIA Hunter platform on extracted clinical samples. A total of 400 RNA samples, extracted from NHS patients nasal-pharyngeal swab samples, were tested with both the VH^6^ platform and the gold standard method: SARS-CoV-2 RT-qPCR.

		Gold standard method: RT-qPCR		
		Positive	Negative	Total
VIDIIA Hunter	Positive	147	0	147
	Negative	3	250	253
	Total	150	250	400

By comparing the RT-qPCR and VIDIIA Hunter platform results, the calculated clinical sensitivity with 95% 2-sided confidence interval was 100% (97.50%–100%) for clinical samples with high viral loads (Ct values up to 25) and 98% (94.28%–99.32%) for all tested clinical samples, the clinical specificity with 95% 2-sided confidence interval was 100% (98.49%–100%), and the positive and negative predicted values were 100% and 98.83%, respectively.

### 3.5 Variants of concern testing

Public Health of England provided our partner hospital (Berkshire and Surrey Pathology Services, Royal Surrey County Hospital) with three chemically inactivated SARS-CoV-2 variants of concern (VOCs). In addition, the VIDIIA Hunter platform was used to test clinical nasal-pharyngeal swab samples throughout the COVID-19 pandemic, during which different VOCs were identified in the clinical samples.

Using the SNPsig^®^ SARS-CoV-2 (EscapePLEX, by Primerdesign Ltd.) RT-qPCR kit to identify the variants of concern, the Berkshire and Surrey Pathology Services confirmed the identification of the three VOCs provided by Public Health England and that, between June and November 2021, the majority of the clinical samples carried the SARS-CoV-2 VOC-21APR-02 B.1.617.2 (Delta). Sequencing also confirmed that the Delta SARS-CoV-2 VOC was predominant in clinical samples between June and November 2021 and that the Omicron SARS-CoV-2 VOC was predominant in clinical samples between December 2021 and April 2022. The three chemically inactivated VOCs as well as 60 clinical samples carrying the Delta variant and 150 clinical samples carrying the Omicron variant were tested using the VIDIIA Hunter platform. Results show that the VIDIIA Hunter platform can detect the main VOCs that were identified in the United Kingdom (as per September 2022), with concordance rate with RT-qPCR: 100% for the Alpha, Beta and Gamma VOCs, 98.33% for the Delta VOC and 98.00% for the Omicron VOC (Table 3).

**TABLE 3:** Variants of concern (VOCs) testing using the VIDIIA Hunter platform. Chemically inactivated SARS-CoV-2 variants of concern (VOCs) and clinical samples were tested with SNPsig^®^ SARS-CoV-2 (EscapePLEX, by Primerdesign Ltd.) RT-qPCR kit, to identify the variants of concern, and with the VIDIIA Hunter. The number of samples detected by VIDIIA Hunter was divided by the number of samples detected by RT-qPCR, to calculate the concordance rate.

Variant type	Samples detected by RT-qPCR	Samples detected by VIDIIA Hunter	Concordance rate (%)
Alpha	8	8	100.00
Beta	8	8	100.00
Gamma	8	8	100.00
Delta	60	59	98.33
Omicron	150	147	98.00

Since the summer of 2022, the test has been subjected to monthly VOCs assurance surveillance. This has been performed *in silico* by comparing the RT-LAMP primer sequences against the variant sequence submissions within the GISAID database. This process allows for the continuous monitoring of the efficiency/specificity of the RT-LAMP test with regards to the genetic changes arising in SARS-CoV-2. To date (September 2023), an average of 15,000 sequences of SARS-CoV-2 per month have been tested using this tool and the percentage of sequences with five or more mismatches within our primers annealing regions has been below 5% of the total number of United Kingdom submitted sequences. The SARS-CoV-2 variants analysed in the *in silico* studies originated from various geographical locations before spreading globally.

## 4 Discussion

In response to the fast-evolving COVID-19 pandemic, a wide range of testing methods have been developed and deployed to identify all infected individuals, including asymptomatic carriers. Rapid and accurate diagnostics are key to control the spread of COVID-19, when paired with an effective track and tracing surveillance system (Peiris et al., 2003; Brouard et al., 2020; Olalekan et al., 2020; Peck, 2020; Walsh et al., 2020; Huff, 2021; Jones et al., 2021). In this study, we developed and validated an affordable, AI-assisted, smart diagnostic platform and an RT-LAMP assay that rapidly and accurately detected SARS-CoV-2 and variants.

The RT-LAMP assay described here recognises the N gene of SARS-CoV-2, which is the most transcribed and highly conserved gene within the Coronaviridae family. When compared to RT-qPCR, the developed assay combined with VIDIIA Hunter diagnostic platform was shown to be reliable, highly specific (100%) and sensitive (100% up to Ct value 25% and 98.0% up to Ct value 35) with a limit of detection of 3 copies of RNA per µL in 20 min and of 1.4 copies of RNA per µL or 1,400 copies/mL in 30 min. The clinical validation of the platform was conducted using a dynamic range of clinically meaningful viral loads: high (Ct values 25 and under), medium (Ct values between 25 and 30), and low (Ct values above 30). The clinical sensitivity was 100% for clinical samples with high viral loads and 98% for all tested clinical samples. Like most molecular diagnostics currently available, the VIDIIA Hunter platform detects samples with high viral loads more accurately than samples with medium and low viral loads. However, the overall sensitivity remains well above 93%, which is the threshold for approval for medical use. Previous studies (Singanayagam et al., 2020; Walker et al., 2021) have demonstrated that samples with high viral loads/low Ct values are correlated with higher infectivity potential, whilst samples with medium and low viral loads are associated lower risks of infectivity at the time of the sampling (in case of early detection of the disease, the risk can increase shortly after sampling). Therefore, the VIDIIA Hunter has the potential to help limit the spread of the disease. To be approved for medical use in the United Kingdom under the United Kingdom Health Security Agency’s Medical Devices (Coronavirus Test Device Approvals, CTDA) (https://www.gov.uk/government/consultations/coronavirus-test-device-approvals-ctda-call-for-evidence/coronavirus-test-device-approvals-ctda-call-for-evidence), COVID-19 PCR and isothermal test needs to have a sensitivity with 95% 2-sided confidence interval entirely above 93% with extraction and above 70% without extraction, and a specificity with 95% 2-sided confidence interval entirely above 97% with extraction and above 93% without extraction (https://www.gov.uk/government/publications/covid-19-test-validation-approved-products, 1st September 2023). Using the data presented here, CE-IVD and MHRA approvals were granted and the VIDIIA Hunter platform has been approved by the CTDA in July 2022. To date (September 2023), a total of 153 COVID-19 tests have been approved by the CTDA, the Optigene COVID-19_Direct Plus (OptiGene Ltd., Horsham, United Kingdom) being the only other RT-LAMP. It is a fluorometric direct test with a sensitivity of 100% below Ct value of 25% and 84% below Ct value 33, a specificity of 100% and a limit of detection of 1,000 copies/mL (Fowler et al., 2021). The U.S. Food and Drug Administration (FDA) also approved a total of 208 COVID-19 molecular tests, with sensitivities ranking between 80% and 100%, specificities between 90% and 100% and LODs between 38 and 100,000 copies/mL (https://www.centerforhealthsecurity.org/covid-19TestingToolkit/molecular-based-tests/current-molecular-and-antigen-tests.html, 1^st^ September 2023). Amongst these molecular FDA approved tests, nine diagnostic devices and a home-test product use RT-LAMP to detect SARS-CoV-2, with sensitivities over 98%, specificities over 91.7% and limits of detection between 0.125 and 20 copies/µL (Oh et al., 2021). Our test is therefore competitive with other SARS-CoV-2 diagnostics approved for medical use. Due to its high sensitivity and specificity, as well as its portability and affordability, the VIDIIA Hunter platform could be used as a primary screening method for SARS-CoV-2, for near patient testing, in resource-limited laboratories or education and healthcare settings. This could have interesting clinical, epidemiological and surveillance applications, by helping with the diagnosis of asymptomatic and mildly symptomatic cases of COVID-19 that have been suggested to be major source of virus propagation within the community (Chan et al., 2020b; Wei et al., 2020). Laboratory and *in silico* data presented here also indicates that the VIDIIA Hunter platform can detect all the SARS-CoV-2 variants of concern that have been circulating in the United Kingdom since the beginning of the pandemic. Since the CTDA approval in the summer of 2022, *in silico* analyses have been performed every month, comparing the RT-LAMP primers sequences with the sequenced genomes of SARS-CoV-2 variants, enabling the continuous monitoring of RT-LAMP test efficiency/specificity with regards to the genetic changes arising in SARS-CoV-2. These *in silico* analyses demonstrated that the VIDIIA Hunter platform can detect all the major SARS-CoV-2 variants that have been circulating in the United Kingdom (from various geographical origins) in the last year. As the N gene is a highly conserved gene, we do not foresee any significant genetic variations in the epitope region of our designed assay, which would be the main limitation to the detection of emerging variants.

The VIDIIA Hunter diagnostic platform, validated in this study, comprises of the Virus Hunter 6 (VH6) device and a smartphone application (VIDIIA companion app for Android). The VH6 device has been developed further from previous studies (Rohaim et al., 2020; Tharmakulasingam et al., 2021). Its thermal profiling was designed and optimised to ensure the stable management of the assay whilst performing the LAMP reaction, using custom aluminium heater blocks. The VH6 is a lightweight, portable, and low-cost device that heats the tubes, takes pictures throughout the amplification, and connects to the VIDIIA companion app for results interpretation. The VH6 is also embedded with an AI, deep learning model that was trained to identify positive, negative, and empty features in the images taken by the device, using a large image dataset generated from 752 clinical samples. A convolutional neural network (CNN) model was chosen for this study, as it is the most commonly applied AI technology in disease diagnosis and has been shown to have a diagnostic performance comparable to medical experts, in image recognition-related fields (Shen et al., 2019). The trained model weights percentage values and generates quantitative data from the colorimetric RT-LAMP pictures. The easy-to-read results can then be consulted on the VH6 device, in the companion app and remotely via the VIDIIA cloud. The AI model worked particularly well in combination with the controlled environment within the VH6, where the background-colour, illumination and position of the PCR strip are constant and controlled, making each image less variable for the reading and interpretation of the results. This allowed to remove layers of complexity due to external factors. Using an AI model allows to remove any subjectivity in the interpretation of the results and can detect smaller colours and turbidity variations than the naked eye. Because the pictures are taken inside the device, the reaction tubes do not need to be opened; therefore, reducing the cross-contamination risk. The VH6 device is also linked to the VIDIIA companion smartphone application (for Android) that works as an easy-to-use interface tool to set-up experiments by configuring the required temperature, time, and volume of images. The smartphone application can also be used by operators to manage and track the patients and samples, from swab collection to registration of samples, and all the way through the testing, thus ensuring that tests are tracible. Results and graphs are displayed on the smartphone application in less than 1 h and the app can automatically notify patients. If patients are notified, they receive an email or SMS with a secure link to the VIDIIA results page. The results page only displays any of three results: Positive, Negative and Inconclusive along with governmental advice. False-positive results might occur due to users’ interpretation, cross-contamination or pH issue and false negative results might happen due to users’ interpretation, inadequately prepared or degraded samples. Errors related to users’ interpretation of results have been overcome using AI. The other issues can be mitigated by following good practices for molecular biology laboratories and using reagents and protocols described in the platform’s instructions for use document. The VIDIIA Hunter platform is an end-to-end testing solution that does not require a computer for results reading and analysis and has been designed to be user-friendly, easy to securely integrate into third-party systems and to assist the testing workflow. The VIDIIA companion app was developed to guide the operator throughout the registration of patients, sample processing, and testing. The app is integrated into the VIDIIA cloud which makes the backup of information seamless and effortless for the end user. The VIDIIA cloud technology includes an integration layer (using an API) which allows easy and secure integration to third party systems such as a Laboratory Information Management System (LIMS). The combination of VIDIIA’s instructions for use, Companion app and cloud creates an ecosystem that is scalable and empowers users to process multiple samples in one or multiple VIDIIA VH6 devices; making the throughput of such system easy to manage. The VIDIIA system allows the decentralisation of testing. Therefore, mass testing can be achieved by taking the testing closer to the root/source of samples. Large numbers of samples can be tested by linking multiple devices.

Other portable molecular testing systems using RT-LAMP have been developed or adapted to respond to the COVID-19 pandemic, such as the fluorometric Optigene COVID-19_Direct Plus test working with the Genie platform [OptiGene Ltd., Horsham, United Kingdom (Fowler et al., 2021)], the colorimetric COV19 qcLAMP test combined with the PEBBLE qcLAMP Platform [BIOPIX DNA TECHNOLOGY P.C. Voutes, Greece, (Papadakis et al., 2022)] and the colorimetric Lucira CHECK-IT COVID-19 Test (Lucira CHECK-IT COVID-19 Test Kit–Instructions for Use (fda.gov), 1st September 2023). The Optigene COVID-19 test obtained comparable performance to the ones presented in this study (Fowler et al., 2021), however the Optigene Genie device is more complex due to the fluorescence detection of the amplification results, therefore more expensive. Furthermore, the Genie device is not connected to a smartphone and requires the use of additional software and hardware to deliver what the VIDIIA Hunter platform can do as part of their offering. The Biopix-T COVID-19 test and its connected platform, PEBBLE qcLAMP, obtained similar sensitivity and specificity to the VIDIIA system (Papadakis et al., 2022), however the results (amplification curves) still require expert knowledge to be interpreted and the test has not be approved by a government agency (such as the FDA or the CTDA). The Lucira CHECK-IT COVID-19 has similar test analytics than the ones presented in this study (Lucira CHECK-IT COVID-19 Test Kit–Instructions for Use (fda.gov), 1st September 2023), however it has been designed for a different use: home testing. For this reason, it can only take one sample at the time and must be disposed of after every test. Portable rapid technologies employing RT-PCR have also been developed to detect SARS-CoV-2 in less than 2 hours, outside of centralised testing labs and several of them have received the FDA and/or the EU approval (Mardian et al., 2021). Amongst them, the Xpert^®^ Xpress SARS-CoV-2 test (Cepheid, Sunnyvale, CA, United States), the CovidNudge (DnaNudge, United Kingdom), the BioFire^®^ FilmArray Respiratory Panel 2.1 (BioFire Diagnostics, Biomérieux, France) and the cobas^®^ Liat^®^ SARS-CoV-2 and influenza A/B test (Roche Molecular Systems, Inc., Pleasanton, CA, United States) have been shown to detect the virus with high sensitivity and specificity, and limits of detection below 1,000 copies/mL. The Xpert^®^ Xpress SARS-CoV-2 test (Cepheid, Sunnyvale, CA, United States) is a cartridge-based PCR assay that rapidly detect the virus using the GeneXpert benchtop system (Wolters et al., 2020). The CovidNudge (DnaNudge, United Kingdom) is a fully automated cartridge-based multiplex RT-PCR targeting seven SARS-CoV-2 gene regions and a host gene, that works with a portable connected device called Nudgebox (Gibani et al., 2020; Yu et al., 2021). The BioFire^®^ FilmArray Respiratory Panel 2.1 (BioFire Diagnostics, Biomérieux, France) uses a closed disposable pouch containing the reagents necessary for sample preparation, RT-PCR targeting the membrane (M) and spike (S) genes of SARS-CoV-2 as well as 22 viral and bacterial respiratory pathogens, and nucleic acid detection. The pouch works with a BioFire System that runs the assay and reports on the presence or absence of each pathogen in the tested sample (Creager et al., 2020). The cobas^®^ Liat^®^ SARS-CoV-2 and influenza A/B test (Roche Molecular Systems, Inc., Pleasanton, CA) is a multiplex RT-PCR targeting two gene regions (ORF1a/b and N) of SARS-CoV-2 and influenza A/B genes, using a cobat analyzer (Hansen et al., 2021). These diagnostics technologies employ expensive components such as microfluidic chips, peristaltic pumps, fluorescent detectors and filters, making them expensive; whilst the VH6 device was built with off-the-shelf components and uses camera technology, making it low-cost. The VIDIIA VH6 device and companion app cost less than £2,000, with a data hosting and service system costing under £2,500 per year, making the VIDIIA Hunter platform 5 to 10 times less expensive than current competitors. Moreover, the GeneXpert system still requires the use of a computer and must be set-up in an air-conditioned room, the CovidNudge system has a low throughput, as it can only test one cartridge at a time and the BioFire FilmArray detects the spike gene that is a hotspot for mutation; some SARS-CoV-2 variants could therefore not be detected by this platform. In comparison to other tests on the market, the VIDIIA Hunter platform is more affordable than most solutions by using less expensive technology, not requiring a centralised approach, and having a small footprint; whilst obtaining comparable test performance, being able to test 6 samples simultaneously in 20 min and detecting all the currently circulating SARS-CoV-2 variants of concern. Moreover, it has been approved for medical use by a government agency, the United Kingdom Health Security Agency’s Medical Devices, and is an end-to-end cloud-based solution that empowers operators with remote and secured access to data without the need of a dedicated computer for results reading.

Based on the results of the study presented here, the VIDIIA Hunter platform has three main advantages. Firstly, it is simple, affordable instrumentation that makes it an attractive solution for near patient testing, in all laboratory and healthcare settings, including low-resources ones. Rapid scaling-up of the device production is foreseen, as it contains off-the-shelves components. Secondly, the platform is connected to a smartphone and cloud, with potential for the VIDIIA cloud to connect to online databases and platforms for surveillance purposes. Finally, the platform is potentially compatible with all reported LAMP assays, developed for the detection of different targets. For example, it could detect other infectious diseases such as sepsis (Barnes et al., 2018; Poirier et al., 2021), tuberculosis (Yadav et al., 2017), dengue (Lau et al., 2015), but also antimicrobial resistance markers (Feng et al., 2021; Poirier et al., 2021), in different types of samples. It could also be used to detect cancer mutations or patient’s cells tolerance to cancer treatment (Wormald et al., 2021). The platform is also not restricted to healthcare diagnostics, it could be used for environmental testing and research and development applications.

In conclusion, the VIDIIA Hunter platform is low-cost, portable (allowing near-patient testing), accurate (high sensitivity and specificity), versatile (can be applied to numerous diseases), can test 6 samples simultaneously, and relies on artificial intelligence for a user-friendly interpretation of the results. The app can be used on or off-line and the data is sent to a cloud when the mobile device connects to the internet, making results easy to access. The diagnostics parameters (temperature, time etc.) are configurable, to be compatible with different assays and the testing devices can be linked to increase throughput. A pilot trial was undertaken and validated the feasibility of the workflow and the affordability of the platform in laboratory testing facilities. Therefore, the VIDIIA Hunter platform could easily be used as a primary screening method for infectious diseases and antimicrobial resistance, for near patient testing, thus helping to control their spread. Further technologies are currently being investigated to facilitate multiplexing.

## Data Availability

The original contributions presented in the study are included in the article/Supplementary Material, further inquiries can be directed to the corresponding authors.

## Supplementary material

SUPPLEMENTARY TABLE S1 Full data set obtained from the 400 clinical samples, as it was submitted to the CTDA for approval.
