# Corrigendum: VIDIIA Hunter: a low-cost, smartphone connected, artificial intelligence-assisted COVID-19 rapid diagnostic platform approved for medical use in the UK

**DOI:** 10.3389/fmolb.2023.1325104

**Published:** 2023-10-31

**Authors:** Aurore C. Poirier, Ruben D. Riaño Moreno, Leona Takaindisa, Jessie Carpenter, Jai W. Mehat, Abi Haddon, Mohammed A. Rohaim, Craig Williams, Peter Burkhart, Chris Conlon, Matthew Wilson, Matthew McClumpha, Anna Stedman, Guido Cordoni, Manoharanehru Branavan, Mukunthan Tharmakulasingam, Nouman S. Chaudhry, Nicolas Locker, Anil Fernando, Wamadeva Balachandran, Mark Bullen, Nadine Collins, David Rimer, Daniel L. Horton, Muhammad Munir, Roberto M. La Ragione

**Affiliations:** ^1^ Department of Comparative Biomedical Sciences, School of Veterinary Medicine, University of Surrey, Guildford, United Kingdom; ^2^ VIDIIA Ltd., Surrey Technology Centre, Guildford, United Kingdom; ^3^ Department of Microbial Sciences, School of Biosciences, University of Surrey, Guildford, United Kingdom; ^4^ Berkshire and Surrey Pathology Services, Molecular Diagnostics, Royal Surrey County Hospital, Guildford, United Kingdom; ^5^ Division of Biomedical and Life Sciences, Faculty of Health and Medicine, The Lancaster University, Lancaster, United Kingdom; ^6^ The Royal Lancaster Infirmary, University Hospitals of Morecambe Bay NHS Foundation Trust, Kendal, United Kingdom; ^7^ GB Electronics (UK) Ltd., Worthing, United Kingdom; ^8^ College of Engineering, Design and Physical Sciences, Brunel University London, Uxbridge, United Kingdom; ^9^ Centre for Vision, Speech and Signal Processing, University of Surrey, Guildford, United Kingdom

**Keywords:** rapid diagnostics, LAMP (loop mediated isothermal amplification), COVID-19, artificial intelligence, infectious diseases

In the published article, there was an error in the **Article title**. Instead of “VIDIIA Hunter diagnostic platform: a low-cost, smartphone connected, artificial intelligence-assisted COVID-19 rapid diagnostics approved for medical use in the UK,” it should be “VIDIIA Hunter: a low-cost, smartphone connected, artificial intelligence-assisted COVID-19 rapid diagnostic platform approved for medical use in the UK”.

The authors apologize for this error and state that this does not change the scientific conclusions of the article in any way. The original article has been updated.

